# Exploring estrogenic activity in lung cancer

**DOI:** 10.1007/s11033-016-4086-8

**Published:** 2016-10-25

**Authors:** Bartosz Kazimierz Słowikowski, Margarita Lianeri, Paweł Piotr Jagodziński

**Affiliations:** Department of Biochemistry and Molecular Biology, Poznan University of Medical Sciences, 6 Święcickiego Street, 60-781 Poznan, Poland

**Keywords:** Estrogen synthesis, Estrogen receptor, Estrogen metabolism, Lung cancer

## Abstract

It is well known that a connection between xenobiotics inhalation, especially tobacco combustion and Lung Cancer development is strongly significant and indisputable. However, recent studies provide evidence indicating that another factors such as, estrogens are also involved in lung carcinoma biology and metabolism. Although the status of estrogen receptors (ER), in both cancerous and healthy lung tissue has been well documented, there is still inconclusive data with respect of which isoform of the receptor is present in the lungs. However according to several studies, ERβ appears to be predominant form. Apart from ERs, estrogens can work through a recently discovered G-coupled estrogen receptor. Binding with both types of the receptors causes a signal, which leads to i.e. enhanced cell proliferation. There are many published reports which suggest that estrogen can be synthesized in situ in lung cancer. Some disturbances in the activity and expression levels of enzymes involved in estrogen synthesis were proved. This suggests that increased amounts of sex-steroid hormones can affect cells biology and be the reason of the accelerated development and pathogenesis of lung cancer. There also exist phenomena which associate estrogenic metabolism and tobacco combustion and its carcinogenic influence on the lungs. Compounds present in cigarette smoke induce the activity of CYP1B1, the enzyme responsible for estrogenic metabolism and synthesis of their cateholic derivatives. These structures during their redox cycle are able to release reactive oxygen species or form DNA adduct, which generally leads to destruction of genetic material. This process may explain the synergistic effect of smoking and estrogens on estrogen-dependent lung cancer development.

## Introduction

The lung cancer (LC) remains the leading cause of cancer death worldwide [1]. Despite current improvements in treatment methods and molecular diagnostics, LC stands as the most frequently appearing type of tumor [1]. The low survival rate of patients suffering from LC is caused mainly by delayed diagnosis and late detection, resulting in identification of disease in advanced stadium and limited treatment options [2]. Clinical classification of LC divides it into two main histopathological types non-small cell lung cancer (NSCLC), recognized in 80% of cases, and small cell lung cancer (SCLC), which occurs less frequently (20%).

LC is very complex disease, related to many environmental, molecular and genetic factors. It is well known that main threats responsible for the development of lung tumors are associated with long-term xenobiotic inhalation, including organic solvent vapors, paints, asbestos, and above all the tobacco combustion [2, 3]. Although the connection between LC development and exposure to cigarette smoke is well documented, current research has also provided evidence that the presence and progression of LC can be affected by gender-dependent factors, especially by estrogens [3–7]. Disturbed expression of the enzymes involved in estrogen synthesis in situ [i.e. aromatase (CYP19A1) and 17-beta-hydroxysteroid dehydrogenase type 1 (HSD17β1) and 2 (HSD17β2)] may lead to changes in intracellular level of 17β-estradiol (E_2_) and thus lead to the enhanced tumorigenesis. Many reports confirm this phenomenon and point to the fact that, LC tissue (compared to histopathologically unchanged material) is characterized by elevated concentrations of E_2_ [7–9]. Additionally, some evidence suggests that E_2_ induces proliferation of several LC cell lines in vitro [7, 8, 10]. This data corresponds to the number of population-based studies which emphasize an inductive effect of sex-hormones on the LC development. The application of hormone replacement therapy is associated with poor survival rate in LC patients, especially post-menopausal women [11, 12]. Lastly, the proven presence of estrogen receptor (ER) in lung tumor tissues (mainly ERβ) suggests that estrogens can exert their effect on cells through ER-mediated effects [13–15].

**Fig. 1:**
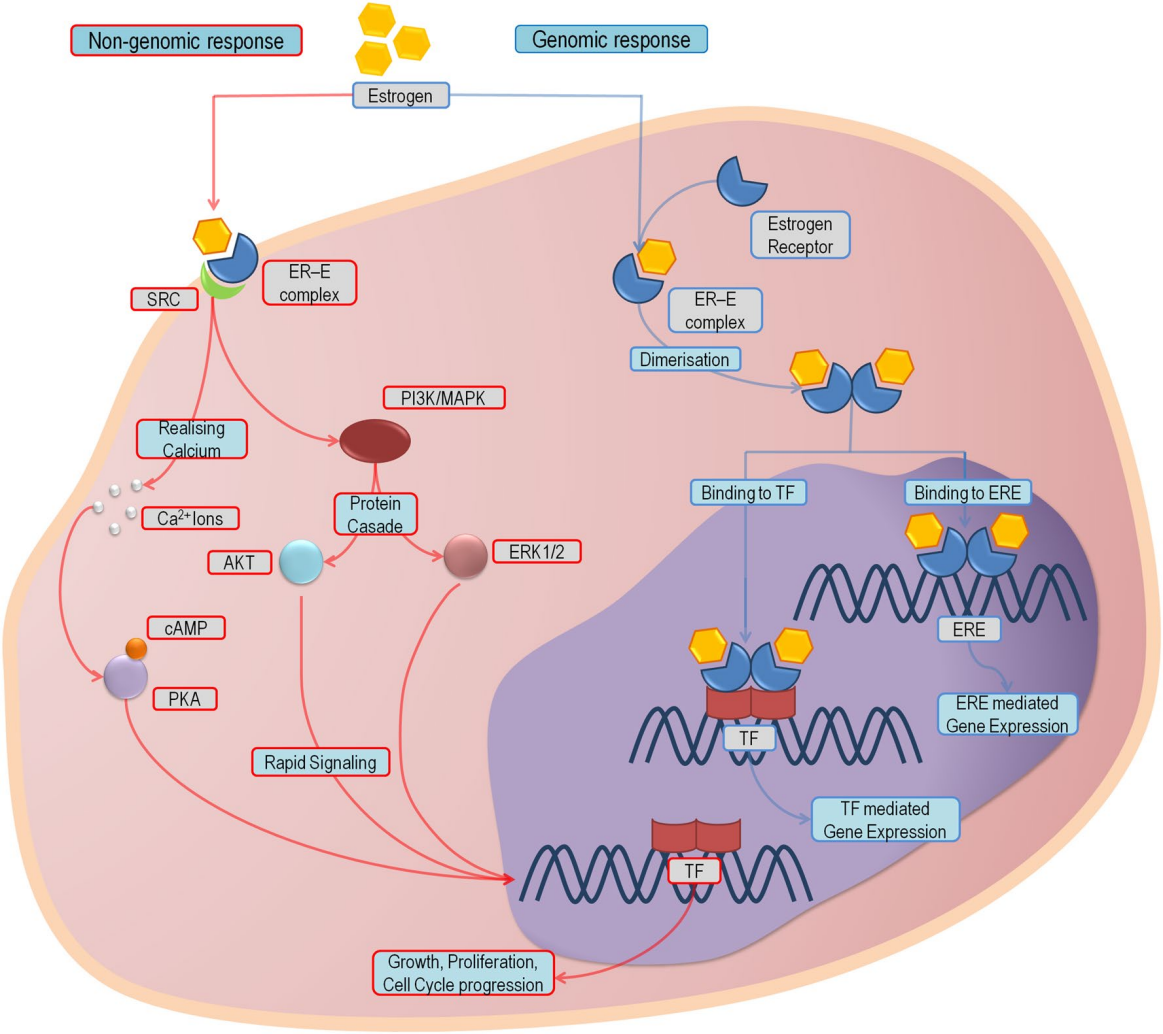
Simplified diagram of estrogen signaling pathways, including non-genomic (*red lines*) and genomic (*blue lines*) response structures (*grey* field) and processes (*cyan* field). *Blue*/*red arrows* indicate the direction of the reaction. *ER* estrogen receptor, *ERE* estrogen responsive elements, *E* estrogen, *TF* transcription factor, *MAPK* mitogen-activated protein kinase, *ERK1*/*2* extracellular regulated kinases, *SRC* proto-oncogene, non-receptor tyrosine kinase, *PI3K* phosphatidylinositide 3-kinase, *Ca* ^*2*+^ calcium ions, *cAMP* cyclic AMP, *PKA* protein kinase A, *AKT* protein kinase B. (Color figure online)

**Fig. 2:**
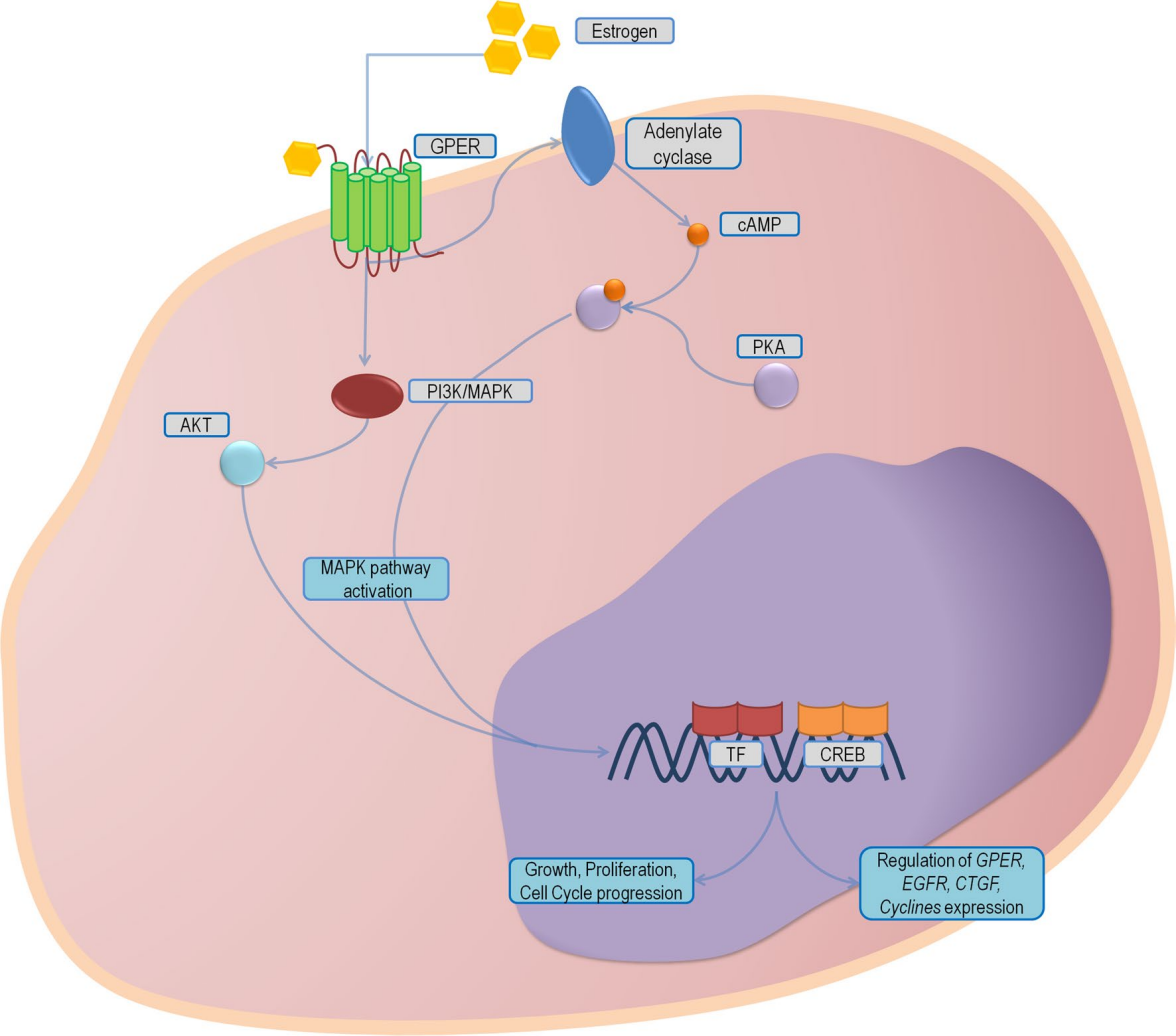
Simplified diagram of GPER response pathways. including structures (*grey* field), processes (*cyan* field). *Blue arrows* indicates the direction of the reaction. *GPER* G-coupled estrogen receptor, *E* estrogen, *MAPK* mitogen-activated protein kinase, *PI3K* phosphatidylinositide 3-kinase, *PKA* protein kinase A, AKT protein kinase B, *EGFR* epidermal growth factor receptor, *CREB* cAMP response element binding protein, *CTGF* connective growth tissue factor, *EGR1* early growth response 1, *TF* transcription factor. (Color figure online)

Estrogens, trough binding with ERs, may affect cells in two different ways: the genomic and the non-genomic manner (Fig. 1). In the non-genomic pathway, estrogens create a complex with cell membrane isoforms of ERs, which trigger an immediate effect, such as activation of non-receptor tyrosine kinases (Src), mitogen activated protein kinases (MAPKs), phosphatidylinositol-3 kinase (PI3K), or releasing intracellular calcium ions (Fig. 1) [16, 17]. In the genomic pathway, estrogens connect to ERs (ERα, ERβ). This action causes the dimerization of ERs, their translocation to the nucleus, and binding with DNA regions known as estrogen response elements (ERE) (Fig. 1). Afterwards, the estrogen-dependent genes are transcribed [16, 18]. Some studies indicate that the recently discovered G-coupled estrogen receptor (GPER, GPR30) can also participate in estrogen response. After connection to the receptor MAPK pathway is activated, which subsequently leads to enhanced cell proliferation. In addition, GPER can also affect the transcription of genes involved in cell cycle and cell growth (Fig. 2) [19–21].

**Fig. 3:**
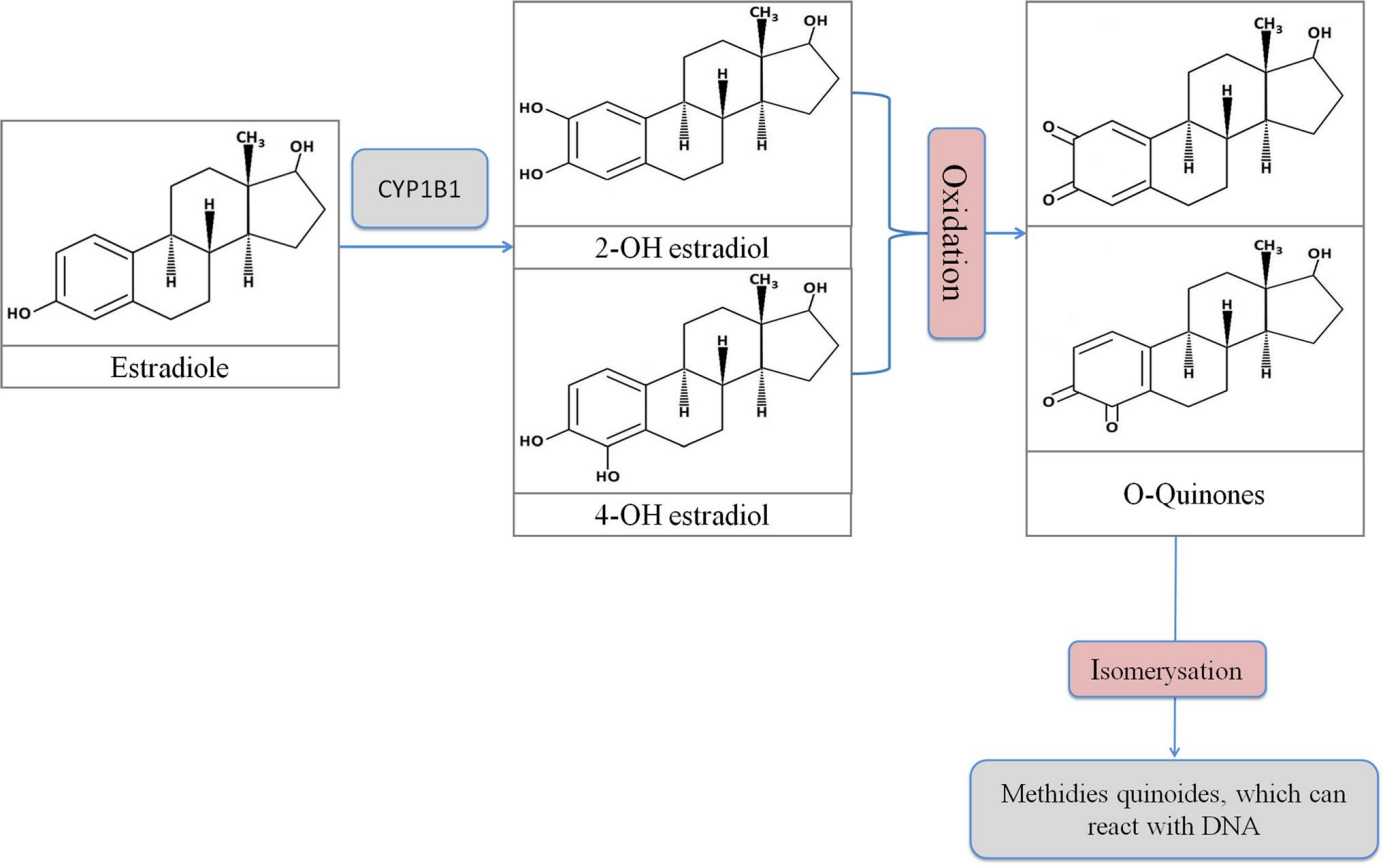
Reaction of O-quinones synthesis including structures (*blue* field) and processes (*pink* field). *Blue arrows* indicates the direction of the reaction. *CYP1B1* cytochrome 450 1B1. (Color figure online)

**Fig. 4:**
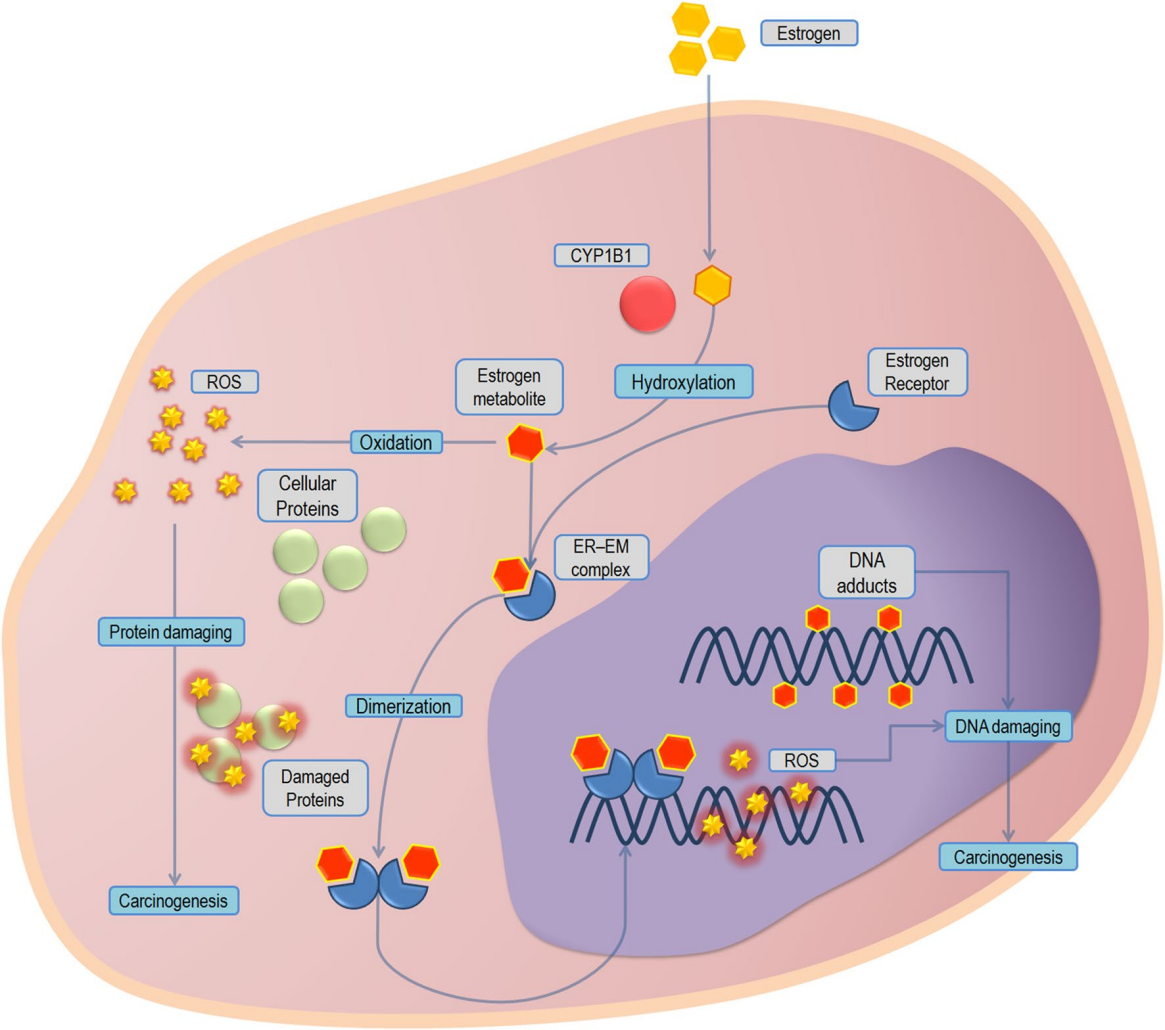
Simplified diagram demonstrated the effect of estrogen metabolites produced by CYP1B1 in cells including structures (*grey* field) and processes (cyan field). *Blue arrows* indicate the direction of the reaction. *EM* estrogen metabolites, *ER* estrogen receptor, *ROS* reactive oxygen species, *CYP1B1* cytochrome P450 1B1. (Color figure online)

By acting through ER, estrogens may also induce cancer development trough formation of genotoxic metabolites such as 4-hydroxyestrogen (4-OHE2), 4-hydroxyestrone (4-OHE_1_) or estrogen’s quinone derivatives [22–24]. This process is strongly connected with cytochrome P450 1B1 (CYP1B1) activity (Fig. 3) which is responsible for the metabolism of E2 as well as present in tobacco smoke carcinogens, to compounds which further transformations results in reactive oxygen species formation (ROS). In addition, the long-lasting tobacco combustion leads to an overexpression of CYP1B1. Subsequently, an increased amount of free radicals is released which may lead to alternated tumorigenesis (Fig. 4) [22–26].

The main aim of this brief review is to focus on possible ways of estrogen action in LC with particular mention on disturbed expression of genes and proteins involved in this process and its association with LC development.

### Estrogen synthesis in normal and malignant lung tissue

**Fig. 5:**
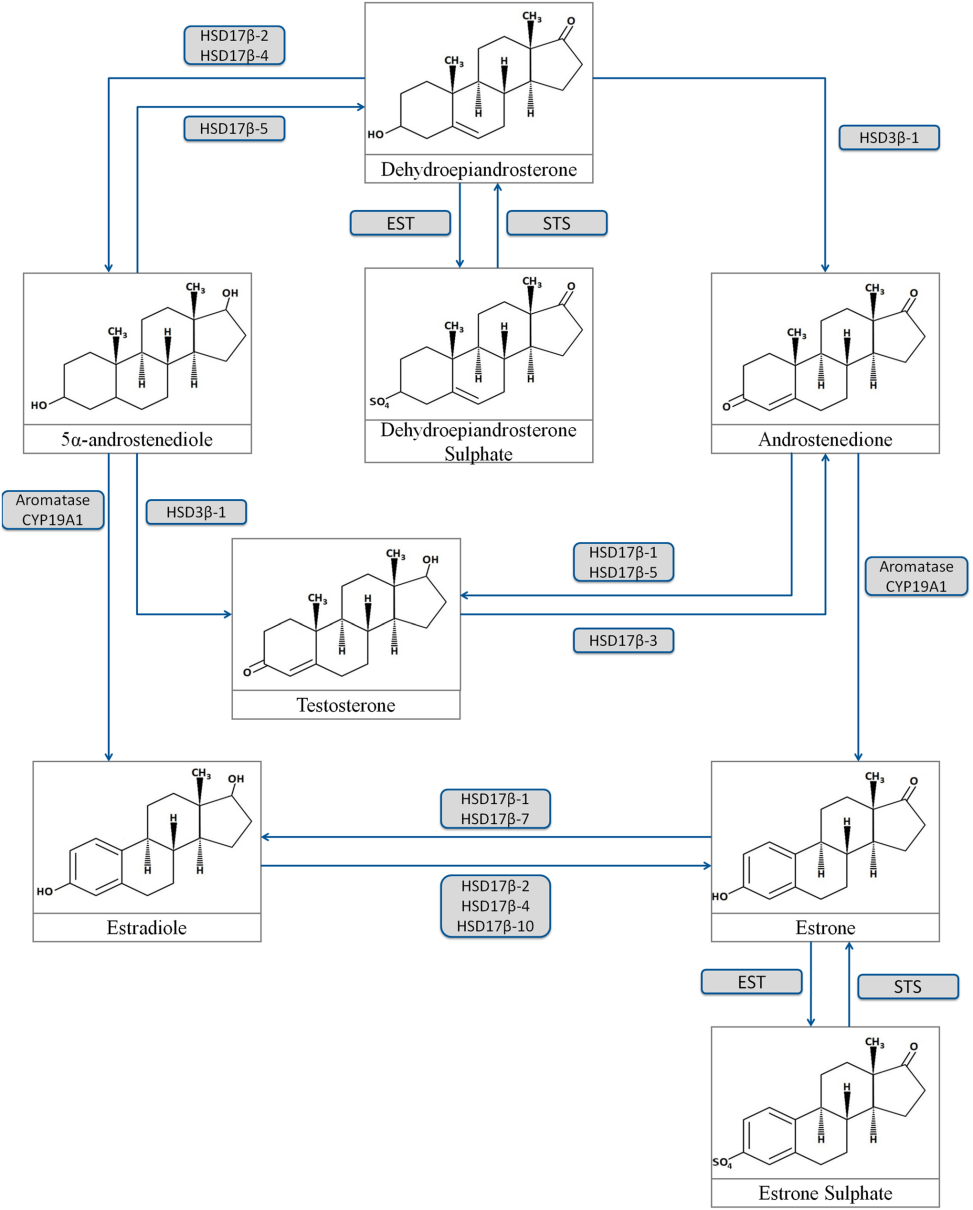
Estrogen metabolism pathway including enzymes (*blue* field) and chemical compounds. *Blue arrows* indicate the direction of the reaction catalyzed by proper enzymes. *HSD17β* hydroxysteroid 17β dehydrogenase, *EST* estrone sulfotransferase, *STS* steroid sufatase. (Color figure online)

There are several important metabolic pathways leading to the formation of estrogens in peripheral tissues. The first one is related to activity of CYP19A1 (cytochrome P450 19A1, aromatase), an steroidogenic enzyme responsible for aromatization of androstenedione and testosterone to estrone (E_1_) or estradiol, respectively [15]. Another crucial protein that participates in local synthesis of estrogen is hydroxysteroid (17-beta) dehydrogenase type 1 (HSD17β1). This particle catalyzes the reduction of E_1_ to the biologically most active E_2_ [8]. Level of peripheral estrogens is also dependent on the activity of sulfatase (STS) and sulfotransferase (EST). STS task is to hydrolyze inactive sulfur derivatives of estrogens to free E_1_, while EST conducts the opposite reaction of binding sulfur moieties to hormones which subsequently leads to their inactivation (Fig. 5) [27].

#### Aromatase (CYP19A1)

CYP19A1 catalyzes the conversion reaction of androstenedione and testosterone to E_1_ and E_2_, respectively (Fig. 5). CYP19A1 is widely expressed in the placenta, ovary, breast, brain and liver [28, 29]. It can also be found in adipose tissue, where it regulates extragonadal estrogen synthesis. This process is also crucial pathway responsible for E_2_ synthesis in men [8, 30, 31]. The CYP19A1 transcript and protein has also been detected in normal and neoplastically changed lung tissues where its expression was found to be higher. Moreover aromatase is also present in metastatic leisons, which are generally characterized by its increased level (compared to primary sites) suggesting carcinogenic influence of estrogens produced in metastatic tissue [14, 32–34]. Through inhibition of aromatase by exemestane in LC cell lines Giannopoulou et al. demonstrated how important CYP19A1 is for cell. Lack of aromatase influenced not only cell migration and invasion but affected cells’ mechanical features too [35].

Many studies confirm that aromatase in LC can be found mainly in the cytoplasm of epithelium cells, which suggests the possibility of producing their own estrogen [29, 36]. These results seem to be similar with data concerning breast cancer, where in situ estrogen synthesis is one of the main factors responsible for tumor growth and its development [28, 37]. However in contrast to breast cancer, in LC aromatase was found mostly in parenchymal cells as compared to stromal site [33]. Nevertheless, by performing experiments of coculturing stromal and carcinoma cells, Miki et al. have shown the stimulating effect of factors secreted by the stroma on CYP19A1 activity. It is very important to note that, the results demonstrated in the same research clearly points also at inductive potential of compounds secreted by NSCLC cells for proliferation and differentiation of stromal cells [33]. This mutual interdependence reveals how many factors have to be considered in the studying of tumor development.

The enhanced activity of CYP19A1 in primary LC tissue has been associated with a high intratumoral concentration of estrogens. This may suggest a potential role of sex-steroid hormones in lung carcinogenesis [13, 14, 33]. The substantial influence of CYP19A1 on growth of the lung tumor was demonstrated by Weinberg et al. The presented data shows the CYP19A1 activity was essentially higher in tumors than in non-histopathologically changed tissue. Additionally in vitro studies showed aromatase impact on lung carcinogenesis. Trough application of the known aromatase inhibitor-anastrozole-enzyme activity has been significantly reduced. This procedure resulted in the expected effect of eradication of lung tumor cells in vitro and inhibited growth of implanted nude mice xenografts [36] Stabile et al. by exposing mice to tobacco carcinogens confirmed this process. Additionally, they have shown that the application of fulvestrant (ER antagonist) enhanced the effect caused by anastrozole [38]. Mah et al. also demonstrated important role of CYP19A1 on the progress of LC. Mice after androstenedione (known substrate of CYP19A1) treatment were presenting more advanced tumor development in comparison to the mice which did not receive the compound. The same effect was observed trough application of E_2,_ thus showing androstenodione may be converted to E2 and support lung carcinogenesis [29]. It is also very important to note that intratumoral levels of CYP19A1 demonstrate a significant association with ER expression and tumor grade. Lower amounts of CYP19A1 in LC are correlated with better prognosis for long term survival (Table 1) [29, 39–41]. All of this data clearly indicate that high level of tumoral aromatase and resultant the high amount of intratumoral estrogen level are essentially connected with LC presence and may affect its progress.

**Table 1:** Summary of available results concerning the status of aromatase in lung carcinoma tissues, including number of patients, year of publication and applied methodology (*IHC* immunohistochemistry, *RT-PCR* reverse transcriptase polymerase chain reaction, *WB* western blot) and obtained results

[References] main authors	Year	No. of patients samples			Methodology	Obtained results
		Total	Female	Male		
[34] Olga K.Weinberg, Diana C. Marquez-Garban	2005	53	33	20	IHC, cell culturing, RT-PCR, WB, animal model	Aromtase was present in NSCLC and lung cancer cell lines. Stronger IHC staining was observed in tumor tissue compared to normal epithelium of bronchioles. Application of aromatase inhibitor results in tumor xenograft suppression and inhibited cell growth
[38] Richard J. Pietras, Diana C. Marquez	2005	?	✓	✗	IHC	Significant expression of aromatase in lung cancer tissue in postmenopausal woman
[29] Vei Mah, David B. Seligson	2007	422	✓	✓	IHC, radioassay	Better survival in >65 years old woman with lower expression of aromatase, especially in those who were characterized by earlier stage of tumor (I/II)
[9] Hiromichi Niikawa, Takashi Suzuki	2008	59	26	33	RT-PCR, liquid chromatography	Intratumoral level of estradiol was significantly connected with aromatase expression. Estradiol enhanced proliferation of expressing aromatase, ERα(+) and ERβ(+) cell lines
[32] Diana C. Márquez-Garbán, Hsiao-Wang Chen	2009	10	?	?	IHC, animal model	Aromatase is expressed in primary and metastatic lesions. Tumor suppression after application of steroidal aromatase inhibitor alone and synergistic effect with cisplatin application
[40] Keiko Abe, Yasuhiro Miki	2009	105	38	67	IHC	ERβ expression was associated with aromatase expression and some clinicopathological features
[33] Yasuhiro Miki, Takashi Suzuki	2010	9	6	3	IHC, RT-PCR	Aromatase is present in carcinoma cells but not in the stromal cells, although some compounds excreted by stroma can affect aromatase activity
[16] Vei Mah, Diana Marquez	2011	377	192	185	IHC	Expression of ERβ with aromatase has predictive value for survival in NSCLC patients
[34] E. Giannopoulou, K.E. Siatis	2014	–	–	–	Cell culturing	Application of exemenstane, demonstrates that modulation of CYP19A1 affects cells migration, invasion and mechanical features

#### Steroid sulfatase (STS) and estrogen sulphotransferase (EST)

STS and EST play an important role in the regulation of steroid hormone synthesis, especially in maintaining balance between active and inactive forms of estrogens. STS conducts desulfulyration reaction of estrone sulfate (E_1_S) and dehydroepiandrosterone sulfate (DHEAS), which subsequently leads to the formation of their active forms (respectively E_1_ and dehydroepiandrosterone; DHEA) [27, 32, 42, 43] (Fig. 5). This reaction, except of aromatization is the main pathway of E_2_ production, since both of aforementioned steroids can be transformed to E_2_ and androstenedione (respectively) and afterwards may enhance the development of sex-hormone dependent tumors, such as breast or prostate cancer [27, 32, 43, 44]. STS activity has been also identified in the liver, testis, adrenal glands, ovary, breast, prostate, skin and brain [27, 32, 43]. EST, on the other hand, usually participates in the inactivation of the E_1_ or E_2_. Binding with sulfates makes the estrogens more soluble and extends their half-life, thus making them ready to use, and when needed they can be easily converted to their active forms by removal of sulfate groups through STS activity [45]. Although the presence of both EST and STS has been demonstrated in sex-hormone dependent cancers, i.e. breast [46] and endometrial cancer [47, 48] there are few studies showing their role in LC. Firstly, data provided by Iida et al. presented status and function of EST and STS in NSCLC [27]. During these investigations, mRNA and protein levels as well as immunoreactivity of STS and EST and concentration of intratumoral estrogens were measured and correlated with some clinicopathological features. Obtained data showed some significant results. STS-positive patients (especially women with adenocarcinoma) were characterized by smaller tumor size, lower cancer cell proliferation and better overall survival. However, the study data showed no statistical differences in mRNA levels between cancerous and histopathologically unchanged tissue, wherein it should be noted that mRNA of *EST* was detected in ~10% examined cases, in contrast to *STS* which was widely detected. On the other hand, the immunoreactivity of STS and EST was marked in 49.5 and 27.8% of the samples, respectively. STS activity was not detected in morphologically normal lung, in contrats to EST which has been weakly indicated in bronchial epithelial cells. Correlation between STS immunoreactivity and intratumoral level of E_1_ or E_2_ was not found, in opposite to EST-immunopositive samples, wherein level of intratumoral E_2_ was significantly higher [27]. These results do not correspond to established by researchers theory about considering the LC as exemplary hormone-dependant tumor i.e. breat cancer [44]. Because of obtained data which was inconsistent with established hypothesis, authors suggest the possibility of different biological roles of STS and EST in NSCLC and emphasize the role of aromatase, which may be more substantial for estrogen synthesis in LC [9, 14, 29, 33–36]. However, in vitro experiments performed by Iida et al. clearly show the possible, important role of STS in LC development. STS-expressing NSCLC cells exposed to E_1_S were characterized by induced proliferation [27]. This phenomenon presents ability of STS to desulfate estrogens to their active forms, allowing them to accelerate cell proliferation, thereby enhancing the carcinogenesis.

Another, more recent work published by Wang et al. demonstrates very important role of EST in regulation of intratumoral estrogen in LC [42]. Researchers tested potential utility of dexamethasone (DEX) as an endocrine therapeutic factor in treating NSCLC. For this purpose they compare activity of DEX and tamoxifen (known antiestrogenic drug) on NSCLC cell lines and tumor xenograft development. Application of DEX resulted in dose-dependent up-regulation of EST in cells, as well as in tumor tissue. DEX exerted antiproliferative effect, inhibited cell migration in vitro and reduced intratumoral level of E_2_. To determine, whether the foregoing phenomena resulted from the increased expression of EST, researchers applied very efficient sulfation inhibitor—triclosan. As expected, application of triclosan reduced the effect caused by DEX, what consequently increased cell survivability, thus showing the major role of EST in LC development and indicating DEX as a potential anti-estrogenic drug in lung tumor treatment.

The results presented by [27, 42] provides important evidence about the role of EST and STS in LC development. Trough the ability of changing balance between active and inactive forms of estrogens, these enzymes can be indicated as the potential prognostic factors or the target proteins in LC therapy.

#### Hydroxysteroid (17-beta) dehydrogenase type 1 (HSD17B1)

Another important steroidogenic protein, HSD17B1, belongs to group of enzymes which catalyze the reversible reaction of E_1_ reduction to its most biologically active metabolite, E_2_ (Fig. 5). An increased expression of HSD17B1 has been noted in many estrogen-dependent cancers i.e. endometrial [49], breast [50] or ovarian tumors [51]. Due to its function, abnormalities in E_2_/E_1_ ratio were also noted in these cancer patients [50, 52]. This made HSD17B1 one of the main factors connected with increased levels of E_2_ in estrogen-dependent cancers [51, 52]. Despite this data, it is presumed that HSD17B1 plays an important role in enhancing the metabolism of E_1_, and has inductive influence on LC development. Niikawa et al. reported, that NSCLC tissues, compared to morphologically normal tissues, are characterized by increased level of E_2_, which has been associated with overexpression of aromatase in these tissues [9]. However, studies which have shown a potential contribution of HSD17B1 in disturbed E_2_/E_1_ ratio were performed by Verma et al. Immunohistological analyses have confirmed the presence of HSD17B1 in the cytoplasm of carcinoma cells in 85% of the investigated samples, while immunostaining of normal bronchial epithelial cells has rarely shown a weak positive signal. The increased immunoreactivity of HSD17B1 in NSCLC tissues was associated with greater tumor grade, and increased level of ERβ and aromatase. Moreover, the enhanced immunointensity of HSD17B1 was correlated with lower E_1_ concentrations in patient’s cancerous tissues. Nonetheless, no distinct differences between HSD17B1 status and increased E_2_ amounts were observed but a significant association with higher intratumoral E_2_/E_1_ ratio was noted. Furthermore, the high immunoreactivity status of HSD17B1 was substantially connected with poor overall survival ratio [53]. The data provided by Drzewiecka et al. confirmed the putative cencerogenic influence of HSD17B1. Western blot immunochemistry and transcript analysis of HSD17B1 showed its statistically significant overexpression in cancerous samples compared to histopathologically unchanged lung tissues, especially among male patients above age 60 who were diagnosed SCC. In addition, in vitro experiments demonstrated that, LC cells are able to transform E_1_ to E_2_ through HSD17B1 activity [8, 54]. These studies clearly show that an increased activity of HSD17B1 could contribute in NSCLC growth and can have inductive influence for estrogen-dependent LC development.

### Estrogen receptor (ER) expression in LC

ERα and ERβ are two different forms of the estrogen receptor, encoded by the *ESR1* and *ESR2* genes, respectively. Both of them have been detected in a variety of hormone responsive tissue, such as breast, ovary and endometrium [13]. In addition, ERs are expressed in the normal lung as well as many NSCLC cells. There are a lot of reports concerning the presence of ER (Table 2) in normal lungs as well as LC, and after many studies which considered ER status, it appears that ERβ is the main functional form of ER in healthy as well as cancerous lung tissue [13–15, 55, 56]. According to Jill M. Siegfried and Laura P. Stabile [13, 15], attention should be paid to research carried out by Brandenberger et al. [109] and Patrone et al. [58]. The first data demonstrates differences between mRNA levels of ERα and ERβ in human tissues during fetal development, showing that ERβ is the only expressed form of ER in the lungs [57]. The second studies used the murine model to present that ERβ in vivo, as well as in vitro, is widely expressed in the epithelium of lungs and is the functional form of the ER. Moreover, the ERβ knockout (−/−), 3 month aged female mouse exhibited a reduced amount of alveoli and surfactant accumulation, which was connected with decreased expression of key regulatory enzymes of surfactant homeostasis and alveoli formation. No such changes were noticed in the ERβ knockout (−/−) male mouse, which was explained by smaller amounts of circulating estrogen [58]. However, Morani et al. showed that female, as well as male, ERβ knockout (−/−), mice lungs at age 5 months were characterized by inefficient alveoli and disturbance in collagen distribution [59], thus displaying that estrogen can play a crucial role in physiological processes of pulmonary diffusion ability and in the development and regeneration of lungs [60]. Moreover microarray data provided by Kerr et al. reveals that the tumoral expression of ERβ is associated with alterations of nearly 500 genes, (while ERα was connected only with 20 genes) which highlighted the importance of ERβ in LC intracellular transformations [61].

**Table 2:** Summary of available results concerning status of ERα and ERβ in lung carcinoma tissues, including number of patients, year of publication and applied methodology (*IHC* immunohistochemistry, *RT-PCR* reverse transcriptase polymerase chain reaction, *WB* western blot) and obtained results

[References] main authors	Year	No. of cases			Methodology	ERα/Erβ status
		Total	Female	Male		
[55] Alfred W. Branderbeger, Meng Kian Tee	1997	–	–	–	RT-PCR, Southern blot	Erβ status confirmed in fetal lungs. No ERα detected
[56] Cesare Patrone, Tobias N. Cassel	2003	–	–	–	Animal model, IHC	Erβ is functional in lungs. Erβ knockout resulted in disturbances in lung homeostasis, which suggests estrogen can play important role in lung development
[57] Andrea Morani, Rodrigo P. A. Barros	2008					
[18] Diana C. Marquez-Garban, Hsiao-Wang Chen	2007	65	45	20	Animal model, IHC	Positive staining Nuclear fraction: 45% for ERα/52% for ERβ Extracellular fraction: 75% for ERα/69% for ERβ
[ER 16] Vei Mah, Diana Marquez	2011	377	142	185	IHC, RT-PCR	ERα: strong signal—nucleus/weak signal—cytoplasm Slight but significant differences between cancerous compared to histologicaly unchanged tissue ERβ: strong signal—nucleus and cytoplasm. More evident differences in expression between examinated tissues. Elevated amounts associated with higher tumor grade. Increased level of ERβ + aromatase predicts worse survival
[54] Hideki Kawai, Akira Ishii	2005	132	56	76	IHC	76% of ERα found in the cytoplasm of poorly or moderate differenciated cancers. Predictor of poor overall survival 51% ERβ found in the nucleus and associated with better overall survival
[69] Mohit Kumar Verma, Yasuhiro Miki	2012	169	66	103	IHC, Cell culturing	Positive staining: 87% samples for ERβ/19% samples for ERα. High cooexpresion of aromatase and ERβ was detected. High ERβ + high aromatase expression predicts worse survival
[62] Ann G. Schwartz, Geoffrey M. Prysak	2005	278	214	64	IHC	Positive ERβ staining: 58.4% for female samples/70% for male samples. No ERα detected. Different nuclear expression of ERβ between cancerous and histopathologically unchanged tissue. More frequent nuclear ERβ expression in adenocarcinoma in male samples, associated with survival status
[63] Birgit Guldhammer Skova, Barbara M. Fischer	2007	104	33	71	IHC	Positive nuclear ERβ and cytoplasmic ERα signal occurred in 69% and 55% samples, respectively. Significantly reduced mortality rate in men ERβ(+) compared to ERβ(−) was noted. No clinicopathological features connected with ERα presence
[66] Hideki Kawai, Akira Ishii	2005	132	55	67	IHC	51.6% samples overexpressed cytoplasmic ERα; ERα linked with poor overall survival. Patients with high level of EGFR associated with elevated amounts of ERα were characterized by worse survival compared to those with low EGFR and ERα
[67] Laura P. Stabile, Sanja Dacic	2010	183	92	91	IHC	ERα and ERβ present in the cytoplasm and nucleus in over 50% samples. Tumors have expressed higher amounts of ERα and ERβ in comparison to histopathologically unchanged tissues. Correlation between ERα, ERβ, Progesterone Receptor and EGFR were examined
[68] Zhuang Luo, Rongrong Wu	2015	2279	✓	✓	Statistical metanalysis	Positive status of ERβ was associated with better survival (except Japan and American population). Overexpression of nuclear form of ERβ predicts better survival
[71] Yoko Omoto, Yasuhito Kobayashi	2001	30	8	22	IHC, WB	Positive staining: 100% ERβ in normal bronchiolar epithelial cells, 67% of tumors were ERβ positive. No expression of ERα was noted. Significant difference in ERβ expression between adenocarcinoma and squamous cell carcinoma, which suggests potential contibution of estrogens in adenocarcinoma development

The ERβ protein has 5 isoforms, though only ERβ-1 is fully functional and able to bind ligand structure, while the rest of them are inactive, however they can form heterodimers with ERβ-1, increasing its transcriptional activity [10, 62]. The ERβ protein is detected, regardless of gender, in primary LC tissues as well as in NSCLC cell lines, both in the cellular cytoplasmatic and nuclear compartments [9, 10, 41, 63–67]. Increased amounts of ERβ can be distinguished in neoplastically changed lung tissue compared to histologically unchanged tissue [16, 65, 68]. Numerous published reports concerning the association between ERβ status and patient survival present different results (Table 2). In most cases, immunohistological analysis of NSCLC samples has indicated an association between ERβ presence and better clinical outcome, especially in men or patients with *EGFR* mutation [63, 65, 68]. Also, an association between positive nuclear ERβ immunostaining and better survival has been observed, while the presence of the cytoplasmic form of ERβ-1 has been indicated as a negative prognosis marker for patient survival [41, 62–69], especially when associated with increased level of aromatase [16]. It is easy to notice that, there exist many investigations concerning ERβ status. Unfortunately each of them considers not enough number of cases to draw any clear conclusion. Because of this, Luo et al. decided to perform a meta-analysis of 2279 cases from 14 rated studies. The obtained results of univariate analysis suggest that ERβ is associated with better overall survival in NSCLC patients, while the multivariate analysis showed no influence of ERβ levels on survival. The provided data confirmed that overexpression of nuclear ERβ is related with better survival, whereas presence of the cytoplasmatic form of ERβ does not predict the survival [70].

There are many reports considering the ERα status in healthy and neoplastically changed lung tissue (Table 2). Most studies show no, or very small amounts of detectable ERα [57, 64, 65, 71–73], though when it was demonstrated more frequently in the cytoplasm than in the nucleus [18, 56, 65, 66], especially in patients with *EGFR* mutation [66, 72]. Immunobloting of cell lines did not detect the full length ERα form, but its 42 and 54 kDa isoforms, which still form a functional protein but characterized by lack of protein amino-terminus [10, 56, 66, 72]. By testing selected agonists of the ERs (α and β), Hershberger et al. proved that ERβ is main receptor responsible for activating both genomic (ERE transcription) and non-genomic pathways (MAPK phosphorylation) [66]. It is also difficult to define the influence of ERα on the overall survival of patients. The existing research indicates that the presence of ERα does not affect survivability [16, 65], nor it is associated with a poor prognosis [66], notably when linked with absence of ERβ [56] or *EGFR* disturbances, especially in Japanese patients with adenocarcinoma [56, 68, 74].

According to this data, ERβ seems to be the primary receptor expressed by LC and control processes, which may lead to estrogen-related carcinogenic actions. ERβ may be the more apparent isoform of the ER in LC (especially NSCLC), thus it could have similar effects on cell growth and signaling as ERα in model estrogen dependent breast cancer [75].

#### G-coupled estrogen receptor (GPER, GPR30)

GPER is present in many different kinds of tissues. Its synthesis has been observed in i.e. ovaries, placenta, testis, uterus, bone narrow, heart, kidneys, liver, and lungs [20, 21]. In response to the cell signal, GPER through a rapid non-genomic mechanism, is able to regulate many physiological functions irrespective of ER classical activity. GPER can induce MAPK, PI3K signaling, affects the regulation of adenylate cyclase and, can activate transcription of *cyclin A, D, E, CTGF* and *EGR1* via EGFR-dependant mechanisms (Fig. 2) [20, 76–79]. Recent studies, concerning the expression and activity of GPER in LC have demonstrated increased amounts of GPER mRNA and protein levels in lung tumors compared to histopathologically unchanged lung tissue [19, 20]. Jala et al. showed elevated transcript and protein amounts of GPER in NSCLC cell lines compared to normal bronchial epithelial cells. In addition, immunohistological staining of human as well as mice LC samples demonstrated an increased activity of GPER in the tumor relative to surrounding non-tumor tissue [20]. These results were confirmed by Liu et al. Moreover, immunohistological analysis of 350 samples showed GPER is more associated with cytoplasmic (80, 49% samples) than the nuclear (53, 05% samples) compartment. Additionally, the expression of cytoplasmic GPER was connected with LC stages IIIA–IV, lymph node metastasis, and poor differentiation of NSCLC. In vitro and animal model studies have shown that the application of E_2_ and selective agonist G1 caused promotion of cell proliferation, migration, and invasion. The opposite effect was obtained by using fulvestrant and G15 inhibitor [19]. Presence of GPER in lung cell allows us to conclude that estrogens may work not only through classic ER. These compounds are able to exert a potential carcinogenic effect through other mechanisms such as GPER activation.

### Smoking and estrogen carcinogenesis

It is well known that the correlation between smoking tobacco and LC (especially squamous cell carcinoma) remains indisputable, while the emerging data suggest the influence of estrogen on LC development. Apart from the genomic or non-genomic response triggered through the connection with ER, estrogen, due to its A-ring-containing structure, can be metabolized by cytochrome P450 enzymes, including cytochrome CYP1B1. CYP1B1 catalyzes hydroxylation at the 2- and 4-position of E_1_ and E_2_, respectively (Fig. 3) [22–24]. Whereas 2-hydroxylated catechol derivatives show no effect, 4-hydroxylated metabolites were found to be carcinogenic factors [80, 81]. In addition, once created, the endogenous catechol estrogens can be oxidized by any enzyme with oxidative activity. This process subsequently leads to the generation of reactive electrophilic estrogen o-quinones and semiquinones, which induce the formation of ROS through redox-cycling process [7, 25, 26, 82]. All of these compounds can affect cells in several, harmful ways. Firstly, the metabolism of O-quinones, through cytochrome P450 activity may indirectly results in the formation of free hydroxyl radicals, which are generally considered as the most harmful oxidizing agents. These undesirable molecules are capable to cause DNA damage, such us single strand breaks, chromosomal aberrations and formation of 8-oxo-dG (8-Oxo-2′-deoxyguanosine)—most frequent DNA oxidative damage. In addition, estrogen quinones and semiquinones, by forming adducts, can directly cause cellular DNA damage, which results in genotoxic effects (i.e. depurination). There are some reports indicating that catechol estrogens, o-quinones or their metabolites are able to bind to the ER, and then subsequently are transported to ERE in the nucleus resulting in DNA mutation and damage caused by free radical emission (Fig. 4) [7, 82–86].

CYP1B1 is a known enzyme responsible for the metabolism of estrogens and procarcinogenic compounds inherent in tobacco smoke (Fig. 3), to carcinogenic derivatives [87]. Several studies have also demonstrated significant association between LC risk and polymorphism of the CYP1B1 [88, 89]. The presence of CYP1B1 has been demonstrated in the lung. Moreover its expression level is different in smokers and non-smokers which has allowed to establish that the CYP1B1 is constantly induced by ongoing tobacco smoke exposure [87, 89–92]. Meireles et al. showed that the *CYP1B1* transcript and protein expression is inducted early during lung tumorigenesis, and its stable increase is maintained over the entire duration of tobacco exposure. There was also a significant amount of E_2_ present in the lung, during this investigation. This phenomenon suggests that CYP1B1 may play a crucial role in tobacco smoke induced carcinogenesis, especially in the presence of estrogens, and provide some evidence that tobacco smoke affects estrogen level within the lungs by altering CYP1B1 [92]. In the comprehensive study by Peng et al. the profile of estrogen metabolites in smokers’ lungs, impact of tobacco smoke and *Cyp1B1* deletion on pulmonary estrogen metabolism were examined. The obtained data confirmed the ability of tobacco smoke compounds to increase the levels of carcinogenic estrogen metabolites, and high levels of carcinogenic estrogen metabolites in female mice were associated with lung tumor promotion by estrogens. Moreover, the deletion of *Cyp1B1* caused a significant drop of carcinogenic estrogen metabolites [7]. According to the aforementioned data, it is presumed that estrogen hormonal environment may synergize with the mutagenicity of tobacco components through the induction of CYP1B1 expression, and may lead to enhanced tumorigenesis.

### Clinical significance of estrogens

LC disease has been intensively over the few past decades. This has allowed researchers to determine, that a history of smoking tobacco is considered as the main harmful factor responsible for its development [5]. However, number of evidence also emphasize the role of gender as the important LC risk factor [4, 93–95]. According to current literature, the risk of all major histopathological types of LC is almost three times higher for smoking women than men, irrespectively on the number of cigarettes smoked per day [93–95]. Additionally, there is a large distinct group of people (approximately 15% men and 53% women) who suffer with LC but have never smoked [1, 96, 97]. It is apparent that, among never-smokers, women also appear to be more vulnerable for LC occurrence (with adenocarcinoma as the most common type) [4, 96]. Because one of the main differences between men and women is the presence of female sex hormones, including estrogens, their commitment in lung cancerogenesis process seems to be suggestive.

Along this line, a significant issue that should be taken into consideration is the use of hormonal replacement therapy (HRT). Studies performed by Adami et al. demonstrated that women who used HRT had slightly elevated risk of developing LC compared to those who were not using HRT. However, the results might be not representative because no adjustment was made for a large group of smoking women [98]. Similarly, a case-control study, performed by Taioli et al. showed that, in a group of never-smoked women, the use of HRT caused no additional risk of LC. However, statistically significant correlation with LC occurrence was observed among the group of smoking women who used HRT. Furthermore, it was found a significant association between HRT use and the incidence of adenocarcinoma. On the other hand, more recent studies demonstrate the opposite effect indicating that, HRT exerts protective action and decreases the risk of LC development [99–103].

Apart from an association with morbidity, estrogens may also affect LC outcomes. Moore et al. found that premenopausal women were characterized by higher frequency of adenocarcinoma occurrence and the cancer was diagnosed at more advanced stadium in comparison to postmenopausal women. Moreover, they establish that postmenopausal women had a slightly decreased death ratio than older men [104]. Despite the fact, that certain important factors, such as age or the use of HRT were omitted during statistical analysis, the results of Moore et al. seem to be confirmed by Ross et al. Because the male concentration of E2 (which is synthesized from testosterone via the aromatase pathway) often occurs at higher levels than in postmenpopausal women [105]. Ross et al. decided to examine the association of E2 amounts with prognosis in male patients with advanced NSCLC. They demonstated that high serum free E2 levels were associated with a decrease in thr survival rate in men corresponding to a shorter survival observed in NSCLC premenopausal women [31].

There are a lot of contradictory studies which present impact of HRT on LC outcome i.e. Ganti et al. and Chlebowski et al. observed that, the lower survival rate among group of women who used hormonal treatment in comparison to patients who did not use it [11, 12]. On the other hand, certain reports show no association between HRT and NSCLC outcomes [106–108]. Certainly, further more extensive studies are needed to elucidate the possible relationship between HRT use and the different type of LC. More detailed data on factors such us, the type of HRT used, gynecologic history, hormonal disturbances, smoking history and age of LC diagnosis is needed to evaluate the impact that estrogens may have on the development of LC, which would be invaluable in disease prognosis and selection of proper therapy.

## Conclusion

Many studies have demonstrated the inductive effect of estrogens on lung carcinogenesis. Growing tumor xenografts and induced cell proliferation clearly show estrogen influence on a cell. A large body of evidence considering gene and protein expression and steroid concentration has demonstrated disturbances in the levels of estrogen and amounts of proper enzymes involved in estrogen synthesis, showing enhanced hormone production in cancer cells. Moreover, the presence of ER, with the dominant ERβ form, demonstrates the possible course of action and influence of estrogens on the cells’ existence. Further, the application of ER antagonists has had an expected effect of inhibition of tumor growth in vivo as well as in vitro, when they exerted a negative effect on LC cell proliferation. The effect of estrogens can be induced not only via ER binding, but also through association with another estrogen-sensitive receptor, GPER, of which the increased activity may lead to enhanced tumorigenesis. Furthermore, the enhanced estrogenic synthesis in LC tissues and its hormonal environment can synergize with the mutagenity of tobaccos smoke components. The combined effect of disturbed estrogenic synthesis in cancer cells and inductive influence of tobacco smoke compounds on estrogen metabolizing enzymes can explain the more aggressive and faster lung tumorigenesis. Synergistic effect of these risk factors is an interesting area of further research.

The amounts of factors which affects the LC development, progression or outcome is enormous, so it is very important to remember that every case of lung tumor is different, just like people are different from each other. This aspects force the detailed molecular examination of the patients which certainly would help with effective and proper treating of the LC.
